# Predictive value of remnant cholesterol level for all-cause mortality in heart failure patients

**DOI:** 10.3389/fcvm.2023.1063562

**Published:** 2023-02-15

**Authors:** Lang Zhao, Xuemei Zhao, Pengchao Tian, Lin Liang, Boping Huang, Liyan Huang, Jiayu Feng, Yuhui Zhang, Jian Zhang

**Affiliations:** ^1^Department of Emergency, China-Japan Friendship Hospital, Beijing, China; ^2^State Key Laboratory of Cardiovascular Disease, Heart Failure Center, Fuwai Hospital, National Center for Cardiovascular Diseases, Chinese Academy of Medical Sciences and Peking Union Medical College, Beijing, China

**Keywords:** remnant cholesterol, heart failure, all-cause mortality, serum lipid, prognosis role

## Abstract

**Background:**

Lower cholesterol levels are associated with increased mortality in heart failure (HF) patients. Remnant cholesterol corresponds to all cholesterol not found in high-density lipoprotein (HDL) and low-density lipoprotein (LDL). The prognostic role of remnant cholesterol in HF remains unknown.

**Objective:**

To reveal the relationship between the baseline remnant cholesterol level and all-cause mortality in HF patients.

**Methods:**

This study enrolled 2,823 patients hospitalized for HF. Kaplan–Meier analysis, Cox regression, C-statistic, net reclassification improvement (NRI), and integrated discrimination improvement (IDI) were used to evaluate the prognostic value of remnant cholesterol for all-cause mortality in HF.

**Results:**

The mortality rate was lowest in the fourth quartile of remnant cholesterol, which had an adjusted hazard ratio (HR) for death of 0.56 [HR: 0.39, 95% confidence interval (CI): 0.46–0.68, *p* < 0.001] relative to the first quartile. After adjustment, a one-unit increase in the level of remnant cholesterol was associated with a 41% decrease in the risk of all-cause mortality (HR: 0.59, 95% CI: 0.47–0.73, *p* < 0.001). A refinement in risk prediction was observed after adding remnant cholesterol quartile to the original model (ΔC-statistic = 0.010, 95% CI: 0.003–0.017; NRI = 0.036, 95% CI: 0.003–0.070; IDI = 0.025, 95% CI: 0.018–0.033; all *p* < 0.05).

**Conclusion:**

Low remnant cholesterol levels are associated with increased all-cause mortality in HF patients. The addition of the remnant cholesterol quartile improved the predictive value over traditional risk factors.

**Clinical Trial Registration:**

ClinicalTrials.gov, Unique Identifier: NCT02664818.

## Introduction

Heart failure (HF) is a clinical syndrome consisting of cardinal symptoms and/or signs due to a structural and/or functional abnormality of the heart that results in elevated intracardiac pressures and/or inadequate cardiac output at rest and/or during exercise (1). The prevalence of HF is approximately 1 ~ 2% worldwide in adults but is increasing due to the aging of the global population (1). Because cholesterol is closely related to nutritional status, irrespective of statin therapy, low levels are associated with increased mortality in patients with HF (2). Indeed, low total cholesterol (TC), high-density lipoprotein cholesterol (HDL-C), and low-density lipoprotein cholesterol (LDL-C) have all been found to be independently associated with increased mortality risk in HF patients (3–7). Remnant cholesterol corresponds to cholesterol not encompassed by HDL and LDL, such as cholesterol associated with very-low-density lipoprotein (VLDL) and intermediate-density lipoprotein (IDL) in the fasting state as well as chylomicron remnants in the non-fasting state (8). Observational studies, and genetic, *in-vitro* and animal studies have suggested a causal association between elevated remnant cholesterol levels, and an increased risk of ischemic heart disease (IHD) (9). Although remnant cholesterol levels correlate with TC, HDL-C, and LDL-C levels, whether remnant cholesterol is a useful indicator for all-cause mortality in patients with HF remains unclear.

In the current long-term follow-up study, we aimed to reveal the prognostic value of the remnant cholesterol for all-cause mortality in HF patients.

## Methods

### Study population

Patients who were admitted to the HF Care Unit (HFCU) of Fu Wai Hospital in Beijing, China, from 2008 to 2018 and diagnosed with HF were enrolled in this study. The diagnosis of each patient was confirmed by 2 cardiologists according to the diagnostic criteria suggested in the “Chinese HF Diagnosis and Treatment Guideline” (10). Data from the first hospitalization were used for any patient who had been hospitalized more than once. Patients who did not complete follow-up, those without cholesterol level data, and patients who underwent heart transplantation or left ventricular assist device (LVAD) implantation during hospitalization were excluded. This study was conducted according to the guidelines of the Declaration of Helsinki, and approved by the ethics review board of Fu Wai Hospital, Beijing, China (approval number: 2018–1,041). Informed written consent was obtained from each patient enrolled in the study.

### Data collection

Baseline information, including age, sex, medical history, clinical manifestations, laboratory values, imaging examination results, and demographics, was obtained from the Electronic Medical Records System of Fu Wai Hospital. Blood samples were drawn from the patient’s antecubital vein after fasting for 8 h and collected into vacuum tubes for measurement on the first morning after admission to the hospital. For all patients, hematological analysis was performed by the clinical laboratory of Fu Wai Hospital. TC levels were measured using the cholesterol oxidase (CHOD-PAP) method, HDL-C levels were measured using the polyethylene glycol-modified enzymes/alpha-cyclodextrin sulfate (PEGME) method, and LDL-C levels were measured using the selective melt method.

### Definition and calculation

Body mass index (BMI) was calculated as body weight (kg) divided by height squared (m^2^). The estimated glomerular filtration rate (eGFR) was calculated using the Modification of Diet in Renal Disease equation using baseline serum creatinine levels (11). Remnant cholesterol was calculated as TC (mmol/L) – HDLC (mmol/L) – LDLC (mmol/L) (8). Hypertension was defined as repeated blood pressure measurements ≥140/90 mmHg at least 3 times on different occasions or a self-reported diagnosis of hypertension (12). Diabetes mellitus (DM) was defined as glycated hemoglobin >6.5%, a fasting serum glucose level ≥ 7.0 mmol/L, random glucose ≥11.1 mmol/L, and/or current diabetes treatment (13). Coronary artery disease (CAD) was defined as angiography-proven coronary stenosis ≥50% of at least one coronary artery (14). HF with preserved ejection fraction (HFpEF) was defined as HF with left ventricular ejection fraction (LVEF) ≥50%. HF with mildly reduced ejection fraction (HFmrEF) was defined as HF with LVEF 41–49%. HF with reduced ejection fraction (HFrEF) was defined as HF with LVEF ≤40% (15).

### Outcomes and follow-up

After discharge, the patients were routinely followed up by outpatient visits or phone calls every 4 weeks for 6 months, every 3 months for the next year, and every 6 months thereafter. The endpoint of this study was time to all-cause mortality. Outcome data for patients discharged from the hospital were collected through routine follow-up, and data on in-hospital mortality were obtained from the Electronic Medical Records System of Fu Wai Hospital.

### Statistical analysis

The statistical analyses were performed using SPSS version 25 (SPSS Inc., Chicago, Illinois) and R version 4.0.2 (The R Foundation, Vienna, Austria). Continuous variables with a normal distribution are presented as the mean ± standard deviation (SD) while continuous variables with a non-normal distribution are presented as the median [first quartile (Q1), third quartile (Q3)]. Categorical variables are presented as numbers (percentages). Continuous variables at baseline were compared using one-way analysis of variance (ANOVA) or the Kruskal–Wallis test according to distribution, followed by Tukey’s post-hoc test to compare the data between two groups. Categorical variables were compared using the chi-square test. Unadjusted Kaplan–Meier curves were constructed, and survival probabilities were compared using the log-rank method. Univariate and multivariate Cox proportional hazards regression were used to evaluate the association of baseline remnant cholesterol levels with time to all-cause mortality. According to our previous studies and guidelines, the selected potential confounders in the multivariable analysis were sex, age, BMI, hypertension, diabetes mellitus, CAD, heart rate, SBP, DBP, hemoglobin, ALB, ALT, AST, TBIL, serum uric, eGFR, hsCRP, logNT-proBNP, LVEF, New York Heart Association (NYHA) Functional Class and pharmacotherapy (1, 10, 15–18). Smooth curve fitting with full adjustment of the covariates was employed to explore the dose–response relationship between remnant cholesterol and the risk of all-cause mortality. Harrell’s C index, net reclassification improvement (NRI), and the integrated discrimination improvement index (IDI) were used for assessing the increase in predictive value when adding remnant cholesterol to a base model including age, sex, NYHA class III/IV vs. I/II, eGFR, and logNT-proBNP. Log transformation was applied when the data were right-skewed. A *p* value <0.05 was considered statistically significant.

## Results

### Baseline clinical characteristics

**Figure 1:**
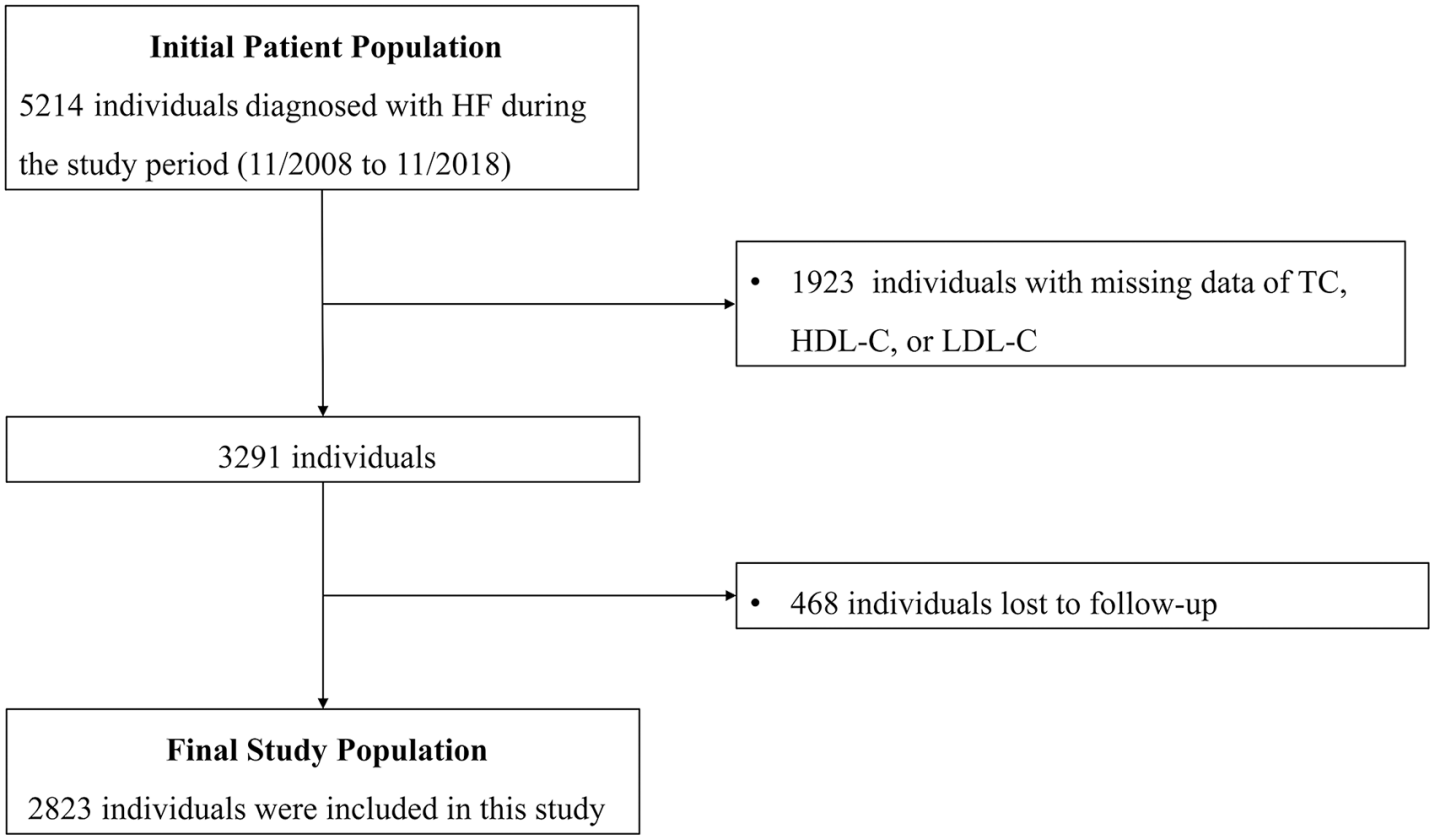
Flow diagram of the screening and enrollment of study participants. HF, heart failure; TC, total cholesterol; HDL-C, high density lipoprotein cholesterol; LDL, low density lipoprotein cholesterol.

As shown in Figure 1, a total of 2,823 HF patients were enrolled in this study. The mean age of the patients was 56.82 ± 15.99 years, and 71.4% of them were men. The prevalence rates of hypertension, DM, and CAD were 49.3, 30.1, and 36.6%, respectively. The mean LVEF of the patients was 39.83 ± 14.32% while HFrEF, HFmrEF, and HFpEF accounted for 54.2, 17.6, and 28.2% of cases, respectively. The mean value of remnant cholesterol at baseline was 0.61 ± 0.38 mmol/L, with a range from 0.01 to 3.99 mmol/L. Patients in higher quartiles of remnant cholesterol level had larger BMI, higher prevalence of hypertension, DM, and CAD and higher blood pressure. Levels of albumin, TC, LDL-C, triglyceride and eGFR were higher while levels of total bilirubin, LVEF, HDL-C, serum uric acid and NT-proBNP were lower in higher quartiles. In addition, smoking prevalence, levels of hemoglobin, ALT and TBIL, type of HF, NYHA Functional Class and rates of digoxin, ACEI/ARB and beta-blocker use also varied among patients with different remnant cholesterol quartiles. The baseline clinical characteristics of all patients, as well as those stratified by quartile of remnant cholesterol level, are shown in Table 1.

**Table 1:** Baseline characteristics by quartiles of remnant cholesterol.

	Total *n* = 2,823	Quartile 1 *n* = 680	Quartile 2 *n* = 714	Quartile 3 *n* = 721	Quartile 4 *n* = 708	*p* value
Male sex, n (%)	2015 (71.4%)	466 (68.5%)	512 (71.7%)	523 (72.5%)	514 (72.6%)	0.292
Age, years	56.82 ± 15.99	56.07 ± 17.14	57.46 ± 16.13	57.69 ± 15.42	56.03 ± 15.19	0.090
BMI, kg/ m^2^	24.52 ± 4.27	23.77 ± 4.17	24.25 ± 4.22	24.62 ± 4.27	25.43 ± 4.25	<0.001
Hypertension, n (%)	1,392 (49.3%)	255 (37.5%)	335 (46.9%)	381 (52.8%)	421 (59.5%)	<0.001
Diabetes mellitus, n (%)	849 (30.1%)	155 (22.8%)	208 (29.1%)	218 (30.2%)	268 (37.9%)	<0.001
Coronary heart disease, n (%)	1,033 (36.6%)	171 (25.1%)	259 (36.3%)	292 (40.5%)	311 (43.9%)	<0.001
Smoking, n (%)	854 (53.1%)	160 (44.9%)	209 (56.3%)	242 (55.0%)	243 (55.0%)	0.006
Drinking, n (%)	621 (38.5%)	128 (36.0%)	147 (39.5%)	173 (39.2%)	173 (39.1%)	0.727
Heart rate, bpm	80.46 ± 18.26	80.25 ± 17.92	81.03 ± 19.64	80.56 ± 19.30	79.97 ± 15.92	0.720
SBP, mmHg	119.34 ± 20.51	116.46 ± 20.87	118.61 ± 20.02	119.64 ± 19.95	122.55 ± 20.82	<0.001
DBP, mmHg	71.87 ± 13.31	70.32 ± 13.54	71.78 ± 13.07	71.88 ± 12.42	73.43 ± 14.02	<0.001
Hemoglobin, g/L	136.86 ± 23.06	134.40 ± 23.77	137.84 ± 21.01	136.76 ± 23.46	138.35 ± 23.77	0.007
ALB, g/L	39.45 ± 5.27	39.14 ± 4.99	39.32 ± 5.22	39.25 ± 5.42	40.08 ± 5.37	0.003
ALT, IU/L	22.00 [14.00,37.00]	21.00 [13.00,35.00]	23.00 [14.00,36.75]	22.00 [14.00,37.00]	24.00 [16.00,40.00]	0.002
AST, IU/L	24.00 [18.00,33.00]	25.00 [19.00,32.00]	23.00 [18.00,34.00]	23.00 [18.00,31.00]	23.00 [18.00,34.00]	0.083
TBIL, μmol/L	25.77 ± 20.96	31.72 ± 21.44	26.15 ± 17.74	23.63 ± 19.77	21.83 ± 23.26	<0.001
Triglyceride, mmol/L	1.54 ± 0.89	0.94 ± 0.32	1.25 ± 0.36	1.56 ± 0.49	2.39 ± 1.24	<0.001
TC, mmol/L	4.13 ± 1.13	3.53 ± 0.95	3.92 ± 0.94	4.24 ± 1.06	4.82 ± 1.16	<0.001
HDL-C, mmol/L	0.99 ± 0.31	1.05 ± 0.35	1.00 ± 0.31	0.97 ± 0.29	0.93 ± 0.27	<0.001
LDL-C, mmol/L	2.54 ± 0.90	2.26 ± 0.82	2.48 ± 0.80	2.62 ± 0.94	2.78 ± 0.96	<0.001
Remnant cholesterol, mmol/L	0.61 ± 0.38	0.22 ± 0.09	0.44 ± 0.05	0.65 ± 0.07	1.11 ± 0.37	<0.001
Serum Uric, μmol/L	459.85 ± 164.11	472.86 ± 168.54	465.65 ± 174.48	452.24 ± 160.24	449.24 ± 151.67	0.021
eGFR, mL/min/1.73 m^2^	74.62 ± 29.07	66.11 ± 27.42	74.02 ± 27.83	76.79 ± 26.43	81.20 ± 32.24	<0.001
hsCRP, mg/L	5.74 ± 4.79	5.06 ± 4.58	6.02 ± 4.96	5.95 ± 4.82	5.91 ± 4.72	<0.001
NT-proBNP, pg./mL	2047.00 [883.50,4608.00]	3186.50 [1351.75,6823.00]	2043.00 [916.75,4977.00]	1669.00 [766.00,3496.00]	1623.50 [694.00,3608.50]	<0.001
LVEF, %	39.83 ± 14.32	41.97 ± 14.83	39.69 ± 13.96	39.11 ± 14.20	38.67 ± 14.10	<0.001
Classification of HF						<0.001
HFrEF, n (%)	1,531 (54.2%)	325 (47.8%)	382 (53.5%)	408 (56.6%)	416 (58.8%)	
HFmrEF, n (%)	497 (17.6%)	117 (17.2%)	142 (19.9%)	125 (17.3%)	113 (16.0%)	
HFpEF, n (%)	795 (28.2%)	238 (35.0%)	190 (26.6%)	188 (26.1%)	179 (25.3%)	
NYHA functional class						<0.001
I	299 (10.6%)	48 (7.1%)	70 (9.8%)	96 (13.3%)	85 (12.0%)	
II	558 (19.8%)	116 (17.1%)	147 (20.6%)	140 (19.4%)	155 (21.9%)	
III	1,255 (44.5%)	330 (48.5%)	319 (44.7%)	314 (43.6%)	292 (41.2%)	
IV	711 (25.2%)	186 (27.4%)	178 (24.9%)	171 (23.7%)	176 (24.9%)	
Pharmacotherapy						
Digoxin, n (%)	1,433 (52.5%)	309 (47.9%)	352 (51.2%)	379 (54.2%)	393 (56.4%)	0.012
ACEI/ARB, n (%)	1,574 (57.7%)	289 (44.8%)	386 (56.1%)	450 (64.4%)	449 (64.4%)	<0.001
Beta-blocker, n (%)	2,390 (87.6%)	522 (80.9%)	603 (87.6%)	621 (88.8%)	644 (92.4%)	<0.001
MRA, n (%)	1941 (71.1%)	424 (65.7%)	486 (70.6%)	505 (72.2%)	526 (75.5%)	<0.001
Loop diuretic, n (%)	2,143 (75.9%)	511 (75.1%)	540 (75.6%)	549 (76.1%)	543 (76.7%)	0.917
Thiazide, n (%)	94 (3.5%)	20 (3.1%)	21 (3.0%)	25 (3.6%)	28 (4.1%)	0.652
VRA, n (%)	53 (2.0%)	15 (2.3%)	9 (1.3%)	17 (2.5%)	12 (1.8%)	0.419

BMI, body mass index; SBP, systolic pressure; DBP, diastolic pressure; ALB, albumin; ALT, alanine aminotransferase; AST, aspartate transaminase; TBIL, total bilirubin; TC, total cholesterol; HDL-C, high density lipoprotein cholesterol; LDL, low density lipoprotein cholesterol; eGFR, estimated glomerular filtration rate; hsCRP, high sensitivity C reactive protein; NT-proBNP, N-terminal pro-brain natriuretic peptide; LVEF, left ventricular ejection fraction; HF, heart failure; HFrEF, heart failure with reduced ejection fraction; HFmrEF, heart failure with mildly reduced ejection fraction; HFpEF, heart failure with preserved ejection fraction; NYHA, New York Heart Association; ACEI, angiotensin-converting enzyme inhibitor; ARB, angiotensin II receptor blocker; MRA, mineralocorticoid receptor antagonist; VRA, arginine vasopressin receptor antagonist. Data presented are mean ± standard deviation, median [Quartile 1, Quartile 3], or n (%).

### Association between baseline low total cholesterol, high-density lipoprotein cholesterol, and low-density lipoprotein cholesterol with all-cause mortality

**Figure 2:**
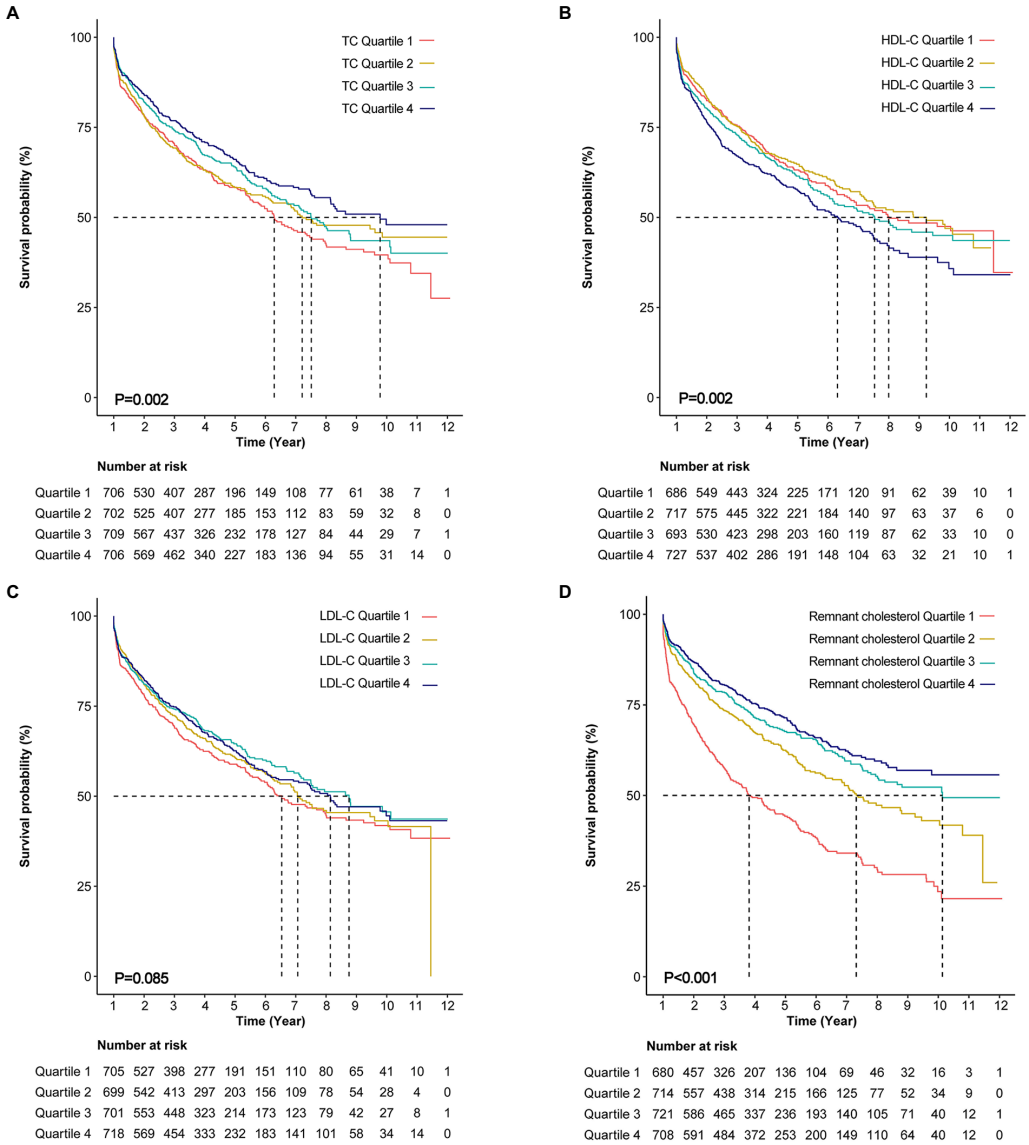
Kaplan–Meier curves for all-cause mortality. **(A)** Kaplan–Meier curves for all-cause death stratified by total cholesterol (TC) quartiles (log-rank, *p* = 0.002). **(B)** Kaplan–Meier curves for all-cause death stratified by high density lipoprotein cholesterol (HDL-C) quartiles (log-rank, *p* = 0.002). **(C)** Kaplan–Meier curves for all-cause death stratified by low density lipoprotein cholesterol (LDL-C) quartiles (log-rank, *p* = 0.085). **(D)** Kaplan–Meier curves for all-cause death stratified by remnant cholesterol (log-rank, *p* < 0.001).

Over a median follow-up of 976 days, all-cause mortality occurred in 1120 (39.7%) patients. The Kaplan–Meier survival curve showed that the patients with the highest quartile of TC had the highest survival probability and the patients with the highest quartile of HDL-C had the lowest survival probability (Figures 2A,B). However, a difference was not found in the survival probability of the patients with different LDL-C quartiles (Figure 2C). After adjusting for age, sex, BMI, comorbidities, laboratory data, NYHA functional class, and pharmacotherapy, the adjusted Cox model showed that only TC was associated with risk of all-cause mortality as continuous variable. Per unit increase in the level of TC was associated with a 7% decrease risk of all-cause mortality [adjusted hazard ratio (HR): 0.93, 0.87–0.99, *p* = 0.028)] (Table 2). When analyzed as a categorical variable, the adjusted HRs for all-cause mortality risk in the fourth quartile of TC compared with the first quartile was 0.7 (95% CI: 0.64–0.35, *p* = 0.014; Table 2). The adjusted HRs for all-cause mortality risk in the fourth quartile of LDL-C was 0.82 (HR 95% CI: 0.68–0.99, *p* = 0.038) compared with the first quartile. Besides, the addition of quartiles of TC, HDL-C, or LDL-C alone did not improve the performance of the base model which included age, sex, NYHA class III/IV vs. I/II, eGFR, and logNT-proBNP for predicting all-cause mortality (Table 3).

**Table 2:** Hazard ratios for all-cause mortality associated with TC, HDL-C, and LDL-C.

	Crude		Adjusted	
	HR (95% CI)	*p* value	HR (95% CI)	*p* value
TC (per 1 unit increase)	0.9 (0.85,0.95)	<0.001	0.93 (0.87,0.99)	0.028
Quartiles of TC				
Quartile 1 (1.16–3.32 mmol/L)	1.00 (Reference)		1.00 (Reference)	
Quartile 2 (3.32–4.03 mmol/L)	0.93 (0.79,1.09)	0.364	0.89 (0.74,1.07)	0.228
Quartile 3 (4.03–4.82 mmol/L)	0.84 (0.72,0.99)	0.037	0.84 (0.69,1.01)	0.066
Quartile 4 (4.82–12.04 mmol/L)	0.73 (0.62,0.87)	<0.001	0.78 (0.64,0.95)	0.014
HDL-C (per 1 unit increase)	1.46 (1.21,1.76)	<0.001	1.24 (0.97,1.57)	0.083
Quartiles of HDL-C				
Quartile 1 (0.04–0.78 mmol/L)	1.00 (Reference)		1.00 (Reference)	
Quartile 2 (0.78–0.95 mmol/L)	0.97 (0.82,1.15)	0.737	0.93 (0.76,1.13)	0.461
Quartile 3 (0.95–1.17 mmol/L)	1.08 (0.91,1.28)	0.368	1.01 (0.82,1.23)	0.939
Quartile 4 (1.17–2.63 mmol/L)	1.29 (1.09,1.52)	0.002	1.07 (0.87,1.32)	0.507
LDL-C (per 1 unit increase)	0.92 (0.86,0.99)	0.022	0.95 (0.88,1.03)	0.194
Quartiles of LDL-C				
Quartile 1 (0.41–1.91 mmol/L)	1.00 (Reference)		1.00 (Reference)	
Quartile 2 (1.91–2.43 mmol/L)	0.91 (0.77,1.07)	0.259	0.89 (0.74,1.07)	0.219
Quartile 3 (2.43–3.05 mmol/L)	0.82 (0.69,0.97)	0.018	0.82 (0.68,0.99)	0.038
Quartile 4 (3.05–10.05 mmol/L)	0.85 (0.72,1)	0.049	0.86 (0.71,1.04)	0.130

TC, total cholesterol; HDL-C, high density lipoprotein cholesterol; LDL, low density lipoprotein cholesterol; HR, hazard ratio; 95% CI, 95% confidence interval. Crude: no adjustment; Adjusted: covariates used for adjustment are sex, age, body mass index, hypertension, diabetes mellitus, coronary heart disease, heart rate, systolic pressure, diastolic pressure, hemoglobin, albumin, alanine aminotransferase, aspartate transaminase, total bilirubin, serum uric, estimated glomerular filtration rate, high sensitivity C reactive protein, log(N-terminal pro-brain natriuretic peptide), left ventricular ejection fraction, New York Heart Association Functional Class and pharmacotherapy.

**Table 3:** C-statistic, δC-statistic, NRI, and IDI of remnant cholesterol for predicting all-cause mortality.

	C-statistic (95% CI)	δC-statistic (95% CI)	*p* value	NRI (95% CI)	*p* value	IDI (95% CI)	*p* value
Original model	0.698 (0.683, 0.714)	–	–	Reference	–	Reference	–
Original model + Quartiles of TC	0.699 (0.684, 0.715)	0.001 (−0.002, 0.003)	0.449	0.000 (−0.022, 0.021)	0.965	0.004 (0.000, 0.007)	0.014
Original model +Quartiles of HDL-C	0.699 (0.683, 0.715)	0.001 (−0.001, 0.002)	0.472	0.008 (−0.009, 0.025)	0.352	0.002 (0.000, 0.036)	0.138
Original model + Quartiles of LDL-C	0.699 (0.683, 0.715)	0.000(−0.002, 0.001)	0.817	0.008 (−0.009, 0.025)	0.360	0.002 (0.000, 0.003)	0.126
Original model + Quartiles of remnant cholesterol	0.709 (0.694, 0.724)	0.010 (0.003, 0.017)	0.005	0.036 (0.003, 0.070)	0.034	0.025 (0.018, 0.033)	<0.001

95% CI, 95% confidence interval; NRI, net reclassification improvement; IDI, integrated discrimination improvement; TC, total cholesterol; HDL-C, high density lipoprotein cholesterol; LDL, low density lipoprotein cholesterol. Original model included traditional risk factors as age, sex, New York Heart Association class III/IV vs. I/II, estimated glomerular filtration rate, and log (N-terminal pro-brain natriuretic peptide).

### Association between baseline remnant cholesterol and all-cause mortality

**Figure 3:**
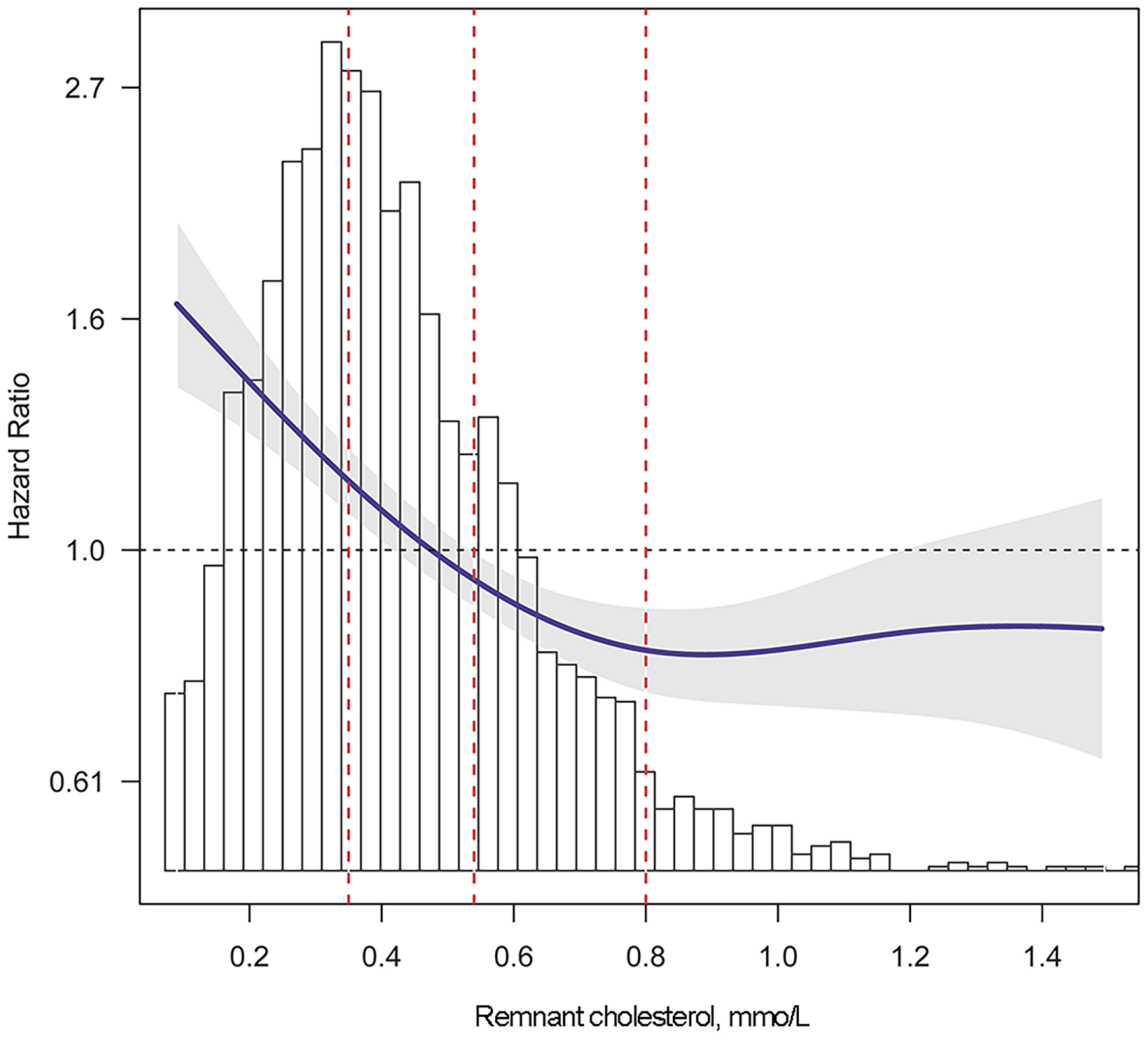
Adjusted risk of all-cause mortality by remnant cholesterol level. Smooth curve fitting was performed to explore the association between remnant cholesterol and the risk of all-cause mortality. Multivariate Cox regression was adjusted for sex, age, body mass index, hypertension, diabetes mellitus, coronary heart disease, heart rate, systolic pressure, diastolic pressure, hemoglobin, albumin, alanine aminotransferase, aspartate transaminase, total bilirubin, serum uric, estimated glomerular filtration rate, high sensitivity C reactive protein, log(N-terminal pro-brain natriuretic peptide), left ventricular ejection fraction, New York Heart Association Functional Class and pharmacotherapy. The solid blue line indicates the adjusted risk of all-cause mortality. The gray shading indicates the 95% confidence interval. The red dotted lines indicate the first, second and third quartile of remnant cholesterol, left to right.

According to Kaplan–Meier survival curve analysis, patients with remnant cholesterol in the lowest quartile had the highest risk of all-cause mortality (Figure 2D). Unadjusted Cox analysis showed an inverse association between baseline remnant cholesterol level and risk of all-cause mortality [HR: 0.39, 95% confidence interval (CI): 0.32–0.48, *p* < 0.001; Table 4]. After adjusting for age, sex, BMI, comorbidities, laboratory data, New York Heart Association (NYHA) functional class, and pharmacotherapy, a one-unit increase in the level of remnant cholesterol was associated with a 41% decrease in the risk of all-cause mortality (HR: 0.59, 0.47–0.73, *p* < 0.001; Table 4). When analyzed as categorical variables, the adjusted HRs for all-cause mortality risk in the second, third and fourth quartiles of remnant cholesterol compared with the first quartile were 0.71 (95% CI: 0.6–0.85, *p* < 0.01), 0.64 (95% CI: 0.53–0.77, *p* < 0.01) and 0.56 (95% CI: 0.46–0.68, *p* < 0.001), respectively (Table 4). The resultant curve fitting with full adjustment displayed a negative correlation between remnant cholesterol and all-cause mortality risk (Figure 3). This result was consistent with the increasing trend across the quartiles in the Cox model (*p* for trend <0.001; Table 4).

**Table 4:** Hazard ratios for all-cause mortality associated with remnant cholesterol.

	Crude		Adjusted	
	HR (95% CI)	*p* value	HR (95% CI)	*p* value
Remnant cholesterol (per 1 unit increase)	0.39 (0.32,0.48)	<0.001	0.59 (0.47,0.73)	<0.001
Quartiles of remnant cholesterol				
Quartile 1 (0.01–0.35 mmol/L)	1.00 (Reference)		1.00 (Reference)	
Quartile 2 (0.35–0.54 mmol/L)	0.57 (0.49,0.67)	<0.001	0.71 (0.6,0.85)	<0.001
Quartile 3 (0.54–0.80 mmol/L)	0.47 (0.40,0.55)	<0.001	0.64 (0.53,0.77)	<0.001
Quartile 4 (0.80–3.99 mmol/L)	0.41 (0.34,0.48)	<0.001	0.56 (0.46,0.68)	<0.001
P for trend		<0.001		<0.001

HR, hazard ratio; 95% CI, 95% confidence interval. Crude: no adjustment; Adjusted: covariates used for adjustment are sex, age, body mass index, hypertension, diabetes mellitus, coronary heart disease, heart rate, systolic pressure, diastolic pressure, hemoglobin, albumin, alanine aminotransferase, aspartate transaminase, total bilirubin, serum uric, estimated glomerular filtration rate, high sensitivity C reactive protein, log(N-terminal pro-brain natriuretic peptide), left ventricular ejection fraction, New York Heart Association Functional Class and pharmacotherapy.

### Remnant cholesterol and all-cause mortality in subgroups

**Figure 4:**
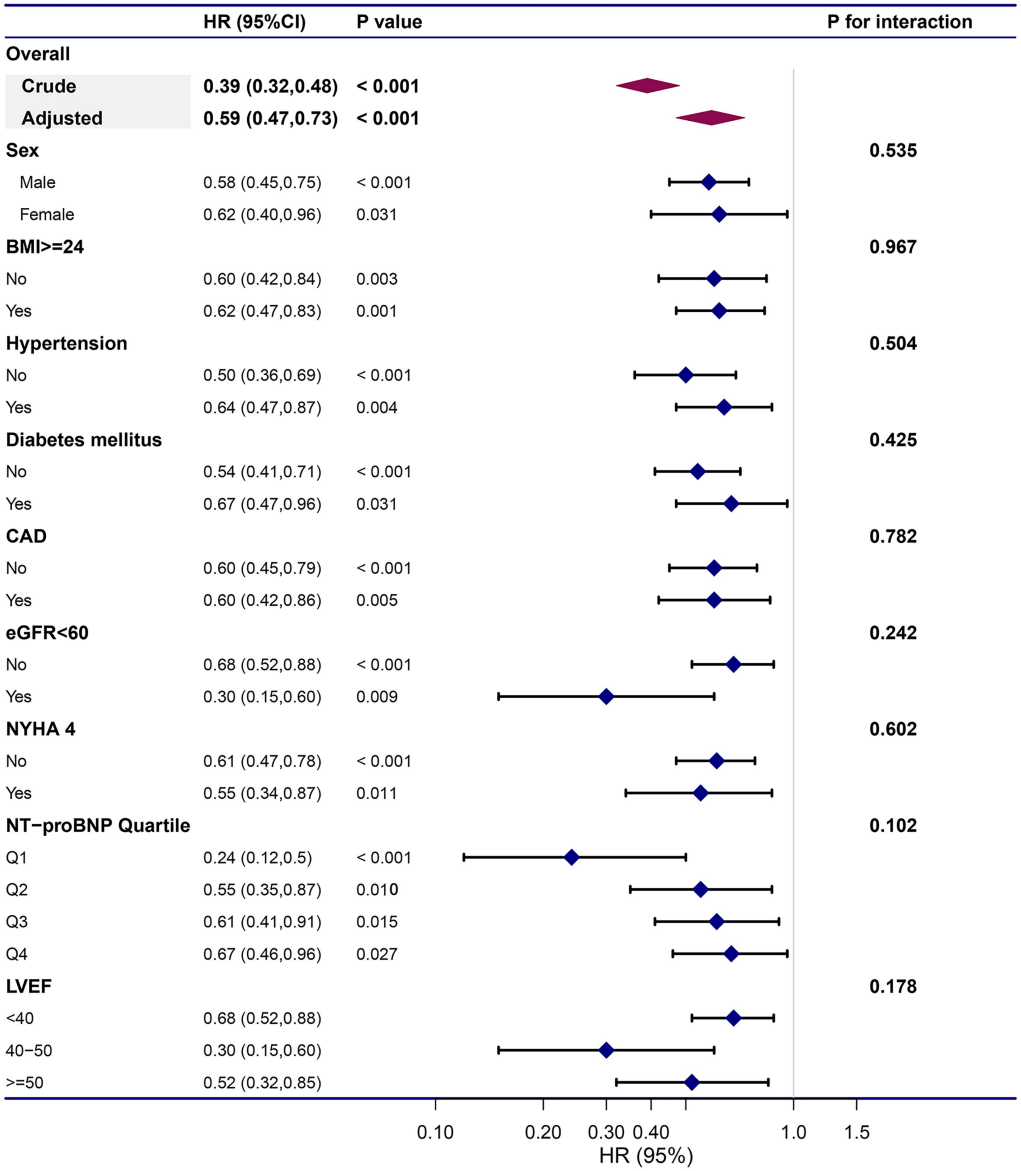
Subgroup analyses on prognostic role remnant cholesterol on all-cause mortality. The dots and lines indicate the estimates of the hazard ratio of all-cause mortality for per unit increment of remnant cholesterol with the corresponding 95% confidence intervals, respectively. The multivariate Cox regression model was adjusted for sex, age, body mass index, hypertension, diabetes mellitus, coronary heart disease, heart rate, systolic pressure, diastolic pressure, hemoglobin, albumin, alanine aminotransferase, aspartate transaminase, total bilirubin, serum uric, estimated glomerular filtration rate, high sensitivity C reactive protein, log(N-terminal pro-brain natriuretic peptide), left ventricular ejection fraction, New York Heart Association Functional Class and pharmacotherapy, except for the variable that is stratified.

To confirm that the Cox analysis findings were robust to potential confounders, we conducted stratified analysis by subgroups defined by covariates shown to have major roles in affecting mortality risk, including age, sex, BMI, hypertension, DM, CAD, eGFR<60, classification of HF (HFrEF, HFmrEF, or HFpEF), NYHA functional class, and quartile of NT-proBNP (Figure 4). All of these analyses were adjusted for age, sex, BMI, comorbidities, laboratory data, NYHA functional class, and pharmacotherapy, except for the covariate that was stratified. Figure 4 illustrates a highly consistent pattern: the risk for all-cause mortality decreased with a higher remnant cholesterol level, regardless of subgroups (all P for interaction>0.05).

### Predictive value of remnant cholesterol for all-cause mortality

A base model including age, sex, NYHA class III/IV vs. I/II, eGFR, and logNT-proBNP for predicting all-cause mortality achieved a C-statistic of 0.698 (95% CI: 0.683–0.714). A refinement in risk prediction was observed when the quartile of remnant cholesterol was added as the C-statistic of the model increased to 0.709 (95% CI: 0.694–0.724) [ΔC-statistic 0.010 (95% CI: 0.003–0.017), *p* = 0.005]. Compared with the base model, adding the remnant cholesterol quartile improved the net reclassification (NRI = 0.036, 95% CI: 0.003–0.070, *p* = 0.034) and increased the IDI (0.025, 95% CI: 0.018–0.033, *p* < 0.001) for all-cause mortality (Table 3).

## Discussion

In this study, we investigated the association of remnant cholesterol with the risk of all-cause mortality and the predictive value of remnant cholesterol for all-cause mortality in HF patients. First, our study found that higher remnant cholesterol levels were independently associated with a lower risk of all-cause mortality. Additionally, the association persisted in the stratification analysis of age, sex, BMI, hypertension, DM, CAD, eGFR, LVEF, NYHA functional class, and NT-proBNP, suggesting that remnant cholesterol has a high predictive value regardless of these covariates. Finally, remnant cholesterol significantly improved the NRI and IDI when added to the base model. The prognostic value of remnant cholesterol provides a new clue to distinguish HF patients with poor prognosis and suggests that the patients with lower remnant cholesterol levels need more intensive and advanced care in hospital and even need strengthened support after discharge. In addition, our study also provides new clinical evidence for blood lipid management in patients with HF.

Remnant cholesterol refers to the cholesterol content of triglyceride-rich lipoproteins, which is composed of chylomicron remnants, VLDL, and IDL in the non-fasting state and VLDL and IDL in the fasting state. Research on the methods for measuring remnant cholesterol has been carried out since the separation of lipoproteins into different classes was first achieved by ultracentrifugation in the 1940s (19). However, it is difficult to create an assay that measures all remnants at the same time because apolipoproteins and lipids dynamically exchange with other lipoproteins and the composition and formation of chylomicron remnants, VLDL, and IDL differ (20). An alternative to direct measurement is to calculate remnant cholesterol levels as total cholesterol minus HDL-C minus LDL-C if directly measured LDLC is used (8).

Remnant cholesterol calculation has been used in several large-scale studies, and remnant cholesterol was found to be associated with worse cardiovascular outcomes. In the PREDIMED (Prevención con Dieta Mediterránea) trial, remnant cholesterol was found to be associated with major adverse cardiovascular events (MACEs) in subjects with overweight or obesity at high cardiovascular risk (21). Furthermore, the FAVORIT study demonstrated that remnant cholesterol is associated with CAD and all-cause mortality in long-term kidney transplant recipients (22), and higher remnant cholesterol levels were significantly associated with worse prognosis in DM and pre-DM patients with CAD in a multicenter prospective study (23). In contrast with these findings, our study found that higher remnant cholesterol levels were independently associated with a lower risk of all-cause mortality and this finding was robust in stratified analysis by subgroups. There are possible mechanistic explanations for the inverse association between remnant cholesterol and all-cause mortality in HF patients. First, the blood lipid levels were lower in the HF patients in our study compared with patients in the above studies. The level of TC, HDL-C, and LDL-C was 4.13 ± 1.13 mmol/L, 0.99 ± 0.31 mmol/L, and 2.54 ± 0.90 mmol/L, respectively. HF is a metabolically demanding condition in which resting energy consumption increases (24). HF patients in our study experienced chronic energy deficiency whereas the patients with CAD and DM may be troubled by the energy surplus. Second, the heart produces a large amount of adenosine triphosphate (ATP) required to maintain systolic function. There are two main sources of ATP production: mitochondrial oxidative phosphorylation and glycolysis. A total of 95% of the ATP required by the myocardium is provided by mitochondrial oxidative phosphate, and the remaining 5% is provided by glycolysis (25). The main energy sources of the heart are fatty acids, lactic acid, glucose, ketones, and amino acids. Among them, fatty acyl CoA, the main metabolite of fatty acids, is an important substrate for the heart to produce ATP (24). Because cardiomyocytes have a low ability to store these energy substrates, triglycerides must be continuously obtained from the blood and hydrolyzed to generate free fatty acids for energy. VLDL carrying remnant cholesterol is also important lipoprotein for transporting triglycerides to cardiomyocytes. As a result, we hypothesized that patients with higher levels of serum remnant cholesterol had more VLDL transporting triglycerides in the serum, which could provide more adequate energy to cardiomyocytes, and therefore the long-term prognosis of the patients was better. Third, per 1 mmol/L higher level of remnant cholesterol was found to be associated with a 37% (95% CI: 35–39) higher C-reactive protein level (26). Infection is an important factor affecting the prognosis of patients with HF (1). After infection, C-reactive protein can enhance the phagocytosis of phagocytic cells by complement activation and play a critical role in innate host defense (27). Therefore, we speculate that HF patients with higher levels of serum remnant cholesterol may have a higher capacity to defend against the risk of infection. However, we believe that there may be other specific protective mechanisms for remnant cholesterol in HF. The mechanisms of remnant cholesterol association with all-cause mortality in HF deserve further investigation. In addition, whether remnant cholesterol should be considered a therapeutic target or just an indicator of a more severe condition needs more research.

Although a trend toward a significant relationship between higher serum lipid and lipoprotein levels and higher survival rates in HF patients has been reported in many studies (2, 3, 28, 29), in our study, after adjusting for significant variables, higher TC levels were found to be associated with all-cause mortality risk in HF patients. Per 1 mmol/L increase in TC level was associated with a 7% decreased risk for all-cause mortality in all HF patients. However, the addition of quartiles of TC alone did not improve the performance of the original model. This result suggests that it is important to identify which subclasses of cholesterol have the dominant influence on HF and are reliable indicators for HF prognosis.

The “HDL hypothesis” that raising HDL-C would reduce MACE and mortality has been proposed since 1977, when the Framingham cohort demonstrated a strong and inverse association between HDL-C and cardiovascular risk (30). Similar results were found in other clinical research focusing on patients with HF (5, 31). Nonetheless, this hypothesis was challenged after practicable methods to quantify each subclass of HDL were proposed (6, 7, 32–34). Moreover, neither proprotein convertase subtilisin/kexin type 9 (PCSK9) inhibitor nor statins appear to have a favorable effect on clinical outcomes in patients with HF (35–37). In our study, HDL-C and LDL-C levels were not found to be associated with all-cause mortality risk in HF patients. This result suggests that it may not be enough to focus only on the HDL-C or LDL-C levels when investigating the effect of cholesterol on the prognosis of HF and that cholesterol in VLDL and IDL may play an important role in the prognosis of HF.

### Strengths and limitations

Our study has several strengths. First, our study reveals for the first time a significant inverse association between remnant cholesterol and all-cause mortality in HF. Second, our study enrolled a broad range of HF patients, including those with and without CAD or DM and those with HFrEF, HFmrEF, or HFpEF. As a consequence, our results are generalizable and suggest that the risk-stratifying ability of remnant cholesterol is applicable to a wide range of HF patients. Third, subgroup analysis revealed a highly consistent pattern, demonstrating the robustness of our findings. Fourth, direct LDL-C measurement was used in the calculation of remnant cholesterol. The direct LDL-C assay is more accurate and provides more clinical information than the Friedewald equation [LDL-C simply equals TG (mmol/L)/2.2]. Fifth, the prognostic value of remnant cholesterol sheds new light on distinguishing patients who should be closely monitored and receive more intensive levels of care and provides new clinical evidence on the management of blood lipids in HF patients.

Our study also has some limitations. First, it was a single-center, retrospective, observational study, and some variables that might have influenced the findings were not measured at baseline. Additionally, the remnant cholesterol concentration was only collected at admission; thus, the association of changes in remnant cholesterol with outcomes could not be investigated. Moreover, as cause-specific mortality was not available, only all-cause mortality was taken into consideration.

## Conclusion

In conclusion, this single-center, retrospective study shows for the first time that the serum level of remnant cholesterol at baseline is an independent predictor of all-cause mortality among hospitalized HF patients. Low admission remnant cholesterol is associated with increased all-cause mortality in HF patients, and adding the quartile of remnant cholesterol improves predictive value over traditional risk factors.

## Data availability statement

The datasets presented in this article are not readily available because the datasets used and analyzed during the current study are available from the corresponding author on reasonable request. Requests to access the datasets should be directed to fwzhangjian62@126.com.

## Ethics statement

The studies involving human participants were reviewed and approved by Ethics review board of Fu Wai Hospital, Beijing, China. The patients/participants provided their written informed consent to participate in this study.

## Author contributions

LZ: conceptualization, formal analysis, investigation, writing—original draft, and writing—review and editing. XZ: formal analysis, data curation, and investigation. PT, LL, BH, LH, and JF: software and investigation. YZ: writing—review and editing, methodology, project administration, supervision, and validation. JZ: writing—review and editing, methodology, project administration, supervision, validation, and funding acquisition. All authors contributed to the article and approved the submitted version.

## Funding

This study was supported by the Key Projects in the National Science and Technology Pillar Program of the 13th Five-Year Plan Period, Beijing, China, Key Projects in the National Science and Technology Pillar Program of the 12th Five-Year Plan Period, Beijing, China, and CAMS Innovation Fund for Medical Science.

## Conflict of interest

The authors declare that the research was conducted in the absence of any commercial or financial relationships that could be construed as a potential conflict of interest.

## Publisher’s note

All claims expressed in this article are solely those of the authors and do not necessarily represent those of their affiliated organizations, or those of the publisher, the editors and the reviewers. Any product that may be evaluated in this article, or claim that may be made by its manufacturer, is not guaranteed or endorsed by the publisher.

## References

[1] McDonagh TA Metra M Adamo M Gardner RS Baumbach A Böhm M. 2021 Esc Guidelines for the diagnosis and treatment of acute and chronic heart failure. Eur Heart J. (2021) 42:3599–726. doi: 10.1093/eurheartj/ehab368, PMID: 34447992

[2] Araújo JP Friões F Azevedo A Lourenço P Rocha-Gonçalves F Ferreira A. Cholesterol--a marker of nutritional status in mild to moderate heart failure. Int J Cardiol. (2008) 129:65–8. doi: 10.1016/j.ijcard.2007.05.026, PMID: 17643521

[3] Horwich TB Hernandez AF Dai D Yancy CW Fonarow GC. Cholesterol levels and in-hospital mortality in patients with acute decompensated heart failure. Am Heart J. (2008) 156:1170–6. doi: 10.1016/j.ahj.2008.07.004, PMID: 19033015

[4] May HT Muhlestein JB Carlquist JF Horne BD Bair TL Campbell BA. Relation of serum Total cholesterol, C-reactive protein levels, and statin therapy to survival in heart failure. Am J Cardiol. (2006) 98:653–8. doi: 10.1016/j.amjcard.2006.03.046, PMID: 16923455

[5] Mehra MR Uber PA Lavie CJ Milani RV Park MH Ventura HO. High-density lipoprotein cholesterol levels and prognosis in advanced heart failure. J Heart Lung Transplant. (2009) 28:876–80. doi: 10.1016/j.healun.2009.04.026, PMID: 19716038

[6] Charach G Argov O Nochomovitz H Rogowski O Charach L Grosskopf I. A longitudinal 20 years of follow up showed a decrease in the survival of heart failure patients who maintained low Ldl cholesterol levels. QJM. (2018) 111:319–25. doi: 10.1093/qjmed/hcy043, PMID: 29733423

[7] Charach G George J Roth A Rogowski O Wexler D Sheps D. Baseline low-density lipoprotein cholesterol levels and outcome in patients with heart failure. Am J Cardiol. (2010) 105:100–4. doi: 10.1016/j.amjcard.2009.08.660 20102899

[8] Nordestgaard BG Langlois MR Langsted A Chapman MJ Aakre KM Baum H. Quantifying atherogenic lipoproteins for lipid-lowering strategies: consensus-based recommendations from Eas and Eflm. Atherosclerosis. (2020) 294:46–61. doi: 10.1016/j.atherosclerosis.2019.12.005, PMID: 31928713

[9] Varbo A Benn M Nordestgaard BG. Remnant cholesterol as a cause of ischemic heart disease: evidence, definition, measurement, atherogenicity, high risk patients, and present and future treatment. Pharmacol Ther. (2014) 141:358–67. doi: 10.1016/j.pharmthera.2013.11.008, PMID: 24287311

[10] Chinese Society of Cardiology, The Editorial Board of Chinese Journal of Cardiology. Chinese guidelines for the diagnosis and treatment of heart failure 2014. Chin J Cardiovasc Med. (2014) 42:98–122. doi: 10.3760/cma.j.issn.0253-3758.2014.02.004 24735621

[11] Ma YC Zuo L Chen JH Luo Q Yu XQ Li Y. Modified glomerular filtration rate estimating equation for Chinese patients with chronic kidney disease. J Am Soc Nephrol. (2006) 17:2937–44. Epub 2006/09/22. doi: 10.1681/asn.2006040368 16988059

[12] Writing Group of 2018 Chinese Guidelines for the Management of Hypertension, Chinese Hypertension League, Chinese Society of Cardiology, Chinese Medical Doctor Association Hypertension Committee, Hypertension Branch of China International Exchange and Promotive Association for Medical and Health Care, Hypertension Branch of Chinese Geriatric Medical Association. 2018 Chinese Guidelines for the management of hypertension. Chin J Cardiovas Med. (2019) 24:24–56. doi: 10.3969/j.issn.1007-5410.2019.01.002

[13] Chinese Diabetes Society. Guideline for the prevention and treatment of type 2 diabetes mellitus in China (2017 edition). Chin J Diab. (2018) 10:4–67. doi: 10.3760/cma.j.issn.1674-5809.2018.01.003

[14] Expert Committee on Rational Drug Use of the National Health and Family Planning Commission of the People's Republic of China, China Pharmacist Association. Guideline for rational medication of coronary artery disease (2nd edition). Chin J Front Med Sci. (2018) 10:1–130. doi: 10.12037/yxqy.2018.06-01

[15] Heart Failure Group for Chinese Society of Cardiology, Chinese Heart Failure Association, The Editorial Board of Chinese Journal of Cardiology. Chinese guidelines for the diagnosis and treatment of heart failure 2018. Chin J Cardiol. (2018) 46:760–89. doi: 10.3760/cma.j.issn.0253-3758.2018.10.004

[16] Feng J Tian P Liang L Chen Y Wang Y Zhai M. Outcome and prognostic value of N-terminal pro-brain natriuretic peptide and high-sensitivity C-reactive protein in mildly dilated cardiomyopathy Vs. dilated cardiomyopathy. ESC Heart Fail. (2022) 9:1625–35. doi: 10.1002/ehf2.13864, PMID: 35243815 PMC9065818

[17] Wang Y Zhang R Huang Y Zhai M Zhou Q An T. Combining the use of amino-terminal pro-B-type natriuretic peptide and B-type natriuretic peptide in the prognosis of hospitalized heart failure patients. Clinica Chimica Acta. (2019) 491:8–14. doi: 10.1016/j.cca.2018.12.025, PMID: 30594544

[18] Zhai M Huang L Liang L Tian P Zhao L Zhao X. Clinical characteristics of patients with heart failure and intracardiac thrombus. Front Cardiovasc Med. (2022) 9:4160. doi: 10.3389/fcvm.2022.934160, PMID: 36277765 PMC9582764

[19] Evaluation of serum lipoprotein and cholesterol measurements as predictors of clinical complications of atherosclerosis; report of a cooperative study of lipoproteins and atherosclerosis. Circulation. (1956) 14:691–742. PMID: 13374848

[20] Varbo A Nordestgaard BG. Remnant lipoproteins. Curr Opin Lipidol. (2017) 28:300–7. doi: 10.1097/mol.0000000000000429 28548974

[21] Castañer O Pintó X Subirana I Amor AJ Ros E Hernáez Á. Remnant cholesterol, not Ldl cholesterol, is associated with incident cardiovascular disease. J Am Coll Cardiol. (2020) 76:2712–24. doi: 10.1016/j.jacc.2020.10.008, PMID: 33272365

[22] Horace RW Roberts M Shireman TI Merhi B Jacques P Bostom AG. Remnant cholesterol is prospectively associated with cardiovascular disease events and all-cause mortality in kidney transplant recipients: the favorite study. Nephrol Dial Transplant. (2022) 37:382–9. doi: 10.1093/ndt/gfab068, PMID: 33760035 PMC8990246

[23] Cao YX Zhang HW Jin JL Liu HH Zhang Y Gao Y. The longitudinal Association of Remnant Cholesterol with cardiovascular outcomes in patients with diabetes and pre-diabetes. Cardiovasc Diabetol. (2020) 19:104. doi: 10.1186/s12933-020-01076-7, PMID: 32631321 PMC7339517

[24] Stanley WC Recchia FA Lopaschuk GD. Myocardial substrate metabolism in the Normal and failing heart. Physiol Rev. (2005) 85:1093–129. doi: 10.1152/physrev.00006.2004 15987803

[25] Honka H Solis-Herrera C Triplitt C Norton L Butler J DeFronzo RA. Therapeutic manipulation of myocardial metabolism: Jacc state-of-the-art review. J Am Coll Cardiol. (2021) 77:2022–39. doi: 10.1016/j.jacc.2021.02.057 33888253 PMC8091273

[26] Varbo A Benn M Tybjærg-Hansen A Nordestgaard BG. Elevated remnant cholesterol causes both low-grade inflammation and ischemic heart disease, whereas elevated low-density lipoprotein cholesterol causes ischemic heart disease without inflammation. Circulation. (2013) 128:1298–309. doi: 10.1161/circulationaha.113.003008, PMID: 23926208

[27] Sproston NR Ashworth JJ. Role of C-reactive protein at sites of inflammation and infection. Front Immunol. (2018) 9:754. doi: 10.3389/fimmu.2018.00754, PMID: 29706967 PMC5908901

[28] Rauchhaus M Clark AL Doehner W Davos C Bolger A Sharma R. The relationship between cholesterol and survival in patients with chronic heart failure. J Am Coll Cardiol. (2003) 42:1933–40. doi: 10.1016/j.jacc.2003.07.016 14662255

[29] Greene SJ Vaduganathan M Lupi L Ambrosy AP Mentz RJ Konstam MA. Prognostic significance of serum Total cholesterol and triglyceride levels in patients hospitalized for heart failure with reduced ejection fraction (from the Everest trial). Am J Cardiol. (2013) 111:574–81. Epub 2012/12/05. doi: 10.1016/j.amjcard.2012.10.042, PMID: 23206923

[30] Gordon T Castelli WP Hjortland MC Kannel WB Dawber TR. High density lipoprotein as a protective factor against coronary heart disease: The Framingham study. Am J Med. (1977) 62:707–14. doi: 10.1016/0002-9343(77)90874-9 193398

[31] Zhao Q Li J Yang J Li R. Association of total cholesterol and Hdl-C levels and outcome in coronary heart disease patients with heart failure. Medicine. (2017) 96:e6094. doi: 10.1097/md.0000000000006094, PMID: 28248864 PMC5340437

[32] Degoricija V Potočnjak I Gastrager M Pregartner G Berghold A Scharnagl H. Hdl subclasses and mortality in acute heart failure patients. Clinica Chimica Acta. (2019) 490:81–7. doi: 10.1016/j.cca.2018.12.020, PMID: 30578754 PMC6591134

[33] Hunter WG McGarrah RW 3rd Kelly JP Khouri MG Craig DM Haynes C. High-density lipoprotein particle sub-fractions in heart failure with preserved or reduced ejection fraction. J Am Coll Cardiol. (2019) 73:177–86. doi: 10.1016/j.jacc.2018.10.059 30654890

[34] Charach G Rabinovich A Ori A Weksler D Sheps D Charach L. Low levels of low-density lipoprotein cholesterol: a negative predictor of survival in elderly patients with advanced heart failure. Cardiology. (2014) 127:45–50. doi: 10.1159/000355164, PMID: 24217704

[35] White HD Schwartz GG Szarek M Bhatt DL Bittner VA Chiang CE. Alirocumab after acute coronary syndrome in patients with a history of heart failure. Eur Heart J. (2021) 43:1554–65. doi: 10.1093/eurheartj/ehab804, PMID: 34922353 PMC9020985

[36] Tavazzi L Maggioni AP Marchioli R Barlera S Franzosi MG Latini R. Effect of Rosuvastatin in patients with chronic heart failure (the Gissi-Hf trial): a randomized, double-blind placebo-controlled trial. Lancet. (2008) 372:1231–9. Epub 2008/09/02. doi: 10.1016/s0140-6736(08)61240-4, PMID: 18757089

[37] Kjekshus J Apetrei E Barrios V Böhm M Cleland JG Cornel JH. Rosuvastatin in older patients with systolic heart failure. N Engl J Med. (2007) 357:2248–61. doi: 10.1056/NEJMoa0706201, PMID: 17984166

